# Epstein-Barr virus serology in relation to the outcomes of oral and genital human papillomavirus infections in women during 3-year follow-up

**DOI:** 10.1038/s41598-026-54544-7

**Published:** 2026-05-29

**Authors:** Sanni Rinne, Birgitta Michels, Julia Butt, Kari Syrjänen, Seija Grenman, Tim Waterboer, Stina Syrjänen, Karolina Louvanto

**Affiliations:** 1 Department of Obstetrics and Gynecology, Faculty of Medicine and Health Technology, Tampere University, Elämänaukio 2, Tampere, 33520 Finland; 2 German Cancer Research Center (DKFZ), Heidelberg, Germany; 3 SMW Consultants, Ltd, Kaarina, Finland; 4 Department of Obstetrics and Gynecology, Turku University Hospital, University of Turku, Turku, Finland; 5 Department of Oral Pathology, Institute of Dentistry, Faculty of Medicine, University of Turku, Turku, Finland; 6 Department of Pathology, Turku University Hospital, Turku, Finland; 7 Department of Obstetrics and Gynecology, Tampere University Hospital, Tampere, Finland

**Keywords:** Epstein-Barr virus, Human papillomavirus, Serology, Oral HPV infection, Genital HPV infection, Cancer, Diseases, Immunology, Microbiology, Oncology

## Abstract

Epstein-Barr virus (EBV) and human papillomavirus (HPV) are common oncoviruses that infect similar anatomical sites, persist as latent infections, and contribute to oncogenesis. However, the role of EBV as a cofactor in HPV infection outcomes remains unclear. This study assessed the implications of EBV serology on oral and genital HPV infections in a longitudinal setting. The study included 280 women from the Finnish Family HPV cohort, followed for three years. Oral, genital, and blood samples were collected at baseline and 12-, 24-, and 36-month follow-ups. EBV-IgG antibodies to Zebra, EA-D, EBNA-1, and VCAp18 proteins were analyzed using multiplex serology, and HPV genotyping was performed with the Multimetrix® assay. Among the 280 women, 98.2% (n = 275) were seropositive for at least two EBV antibodies, with stable antibody levels over the follow-up. Self-reported atopy increased the likelihood of high Zebra and VCAp18 antibody levels OR 2.11 (95%CI 1.07–4.17) and OR 2.45 (95%CI 1.24–4.83). No clear associations between EBV serology and longitudinal oral and genital HPV outcomes were found. In conclusion, nearly all women tested positive for EBV antibodies, however, no notable implications between EBV antibody levels and longitudinal outcomes of oral and HPV infection outcomes could be determined.

**Supplementary Information:**

The online version contains supplementary material available at 10.1038/s41598-026-54544-7.

## Introduction

Epstein-Barr virus (EBV), also known as human herpes virus 4 (HHV4) is a gamma-herpesvirus of the *Herpesviridae* family. EBV is a ubiquitous virus that affects nearly 90% of the population before 30 years of age^1^. It is known as the primary cause of infectious mononucleosis, with most symptomatic infections occurring in adolescence and early adulthood, while primary infections acquired in childhood are more frequently asymptomatic^1^. EBV has also been linked with the development of several human malignancies, including Hodgkin’s lymphoma, Burkitt lymphoma, nasopharyngeal carcinoma, and gastric carcinomas^1^. Risk factors of EBV transmission have been linked to high-risk sexual behavior (high number of sexual partners, unprotected sexual intercourse, and not using oral contraceptives)^2,3^, but transmission via saliva (deep kissing) seems to be the only widely accepted transmission mode of EBV in adolescents and young adults^4^. Primary infection can be detected by EBV-IgM serology, while past infection or reactivation of EBV is confirmed by EBV IgG serology^1,5^.

Human papillomaviruses (HPVs) are known to infect the skin as well as epithelial cells of the oral and genital mucosa^6^. Persistent high-risk HPV (HR-HPV) infections are known to be an etiological factor in nearly 99% of cervical cancer cases and 42%-72% of oropharyngeal squamous cell cancers (OPSSC)^7,8^. Despite HPV being a common virus, only few HPV-infected subjects will have natural serological responses to the virus^9^. The presence of HPV antibodies has been linked to HPV DNA persistence^9^, but the true connection between HPV infections and HPV serology is still under debate. In the published reports, the estimates of HPV seroprevalence usually show a variation from 3% to 44%; however, as high as 70% has been reported for some HPV types^10^.

EBV and HPV are both well-known human oncoviruses, yet surprisingly little is known about their possible co-infections. This is unexpected since both viruses infect epithelial cells, often at the same mucosal sites (such as the head and neck and genital tract), and both can establish latent infections that may reactivate. In latent infections, the EBV genome replicates only once with each cell cycle, similar to that of HPV, while during lytic reactivation, the EBV genome replicates to generate a high number of viral genomes, to be packaged into infectious particles for transmission^11^. Interestingly, a 2018 meta-analysis indicated that EBV/HR-HPV co-infection is related to an increased risk of cervical cancer^12^.

In the present study, we determined both EBV seroreactivity and stability of the EBV antibodies in women prospectively followed up for 36 months in the Finnish Family HPV cohort. We also explored the associations of EBV serology with the natural history of HPV infections in this cohort, with special reference to their implicated oncogenic properties^1,13^. We hypothesized that elevated EBV antibody levels would prolong the persisting oral and genital HPV infections during the follow-up.

## Materials and methods

### The Finnish family HPV study

The Finnish Family HPV Study (FFHPV) is a prospective cohort study investigating the dynamics of HPV infection among family members^14^. The study was conducted jointly by the Department of Obstetrics and Gynecology at Turku University Central Hospital and the Institute of Dentistry, Faculty of Medicine, University of Turku, Finland. The recruitment for the study was carried out between 1998 and 2001 at the maternity unit of the Turku University Central Hospital. In total, the study included 329 mothers (mean age 25.5 years), 131 fathers, and their 331 children (two sets of twins). The mothers included were recruited at a minimum of 36 weeks of pregnancy. A questionnaire was provided at baseline that consisted of questions concerning sexual habits, gynecological and obstetric history, smoking and alcohol consumption, and overall health. The questionnaire has been discussed in more detail previously^14^. The original study protocol and its subsequent amendments were approved by the Research Ethics Committee of Turku University Hospital, Finland (#3/1998, #2/2006, 45/180/2010, and 329/2018). The research was conducted in accordance with the Declaration of Helsinki. Informed written consent was obtained from all participants of the study.

### Sampling

Detailed descriptions of the sample collections in the FFHPV study have been published earlier^14^. In brief, oral and genital brush samples were collected at baseline when the women were pregnant, and at 2-, 6-, 12-, 24-, 36-, and 72 months of follow-up. Oral scrapings were collected from the entire buccal mucosa, including the upper and lower vestibular area, using Cytobrush^®^ (MedScan, Malmö, Sweden). The cervical swabs were taken after a Pap smear from the cervical mucosa, with the transformation zone samples being collected in parallel^14^. Blood samples were taken from the women at baseline and 12-, 24-, and 36-months of follow-up. After collection, the blood samples were centrifuged at 1150 g for 10 min (Sorvall GLC-2, DuPont Instrument, Newtown, Connecticut, USA), the serum was divided into three aliquots and stored first at − 20 °C for no longer than 1 week, and then at − 70 °C until delivered to the German Cancer Research Center (DKFZ), Heidelberg, Germany for serological analysis.

### HPV genotyping

The HPV genotyping was performed using a multiplex HPV genotyping kit with modifications (Multimetrix^®^; Progen Biotechnik GmbH) as described earlier^15^. Altogether, 24 low-risk HPV (HPV 6, 11, 42, 43, 44, and 70) and high-risk HPV (HPV 16, 18, 26, 31, 33, 35, 39, 45, 51, 52, 53, 56, 58, 59, 66, 68, 73, and 82) genotypes were identified among the participants of our study.

### EBV and HPV serology

The serum IgG antibody levels to four different EBV proteins: viral capsid antigen p18 (VCAp18), EBV nuclear antigen 1 (EBNA-1), early diffuse antigen complex (EA-D), and BZLF1-encoded replication activator protein of EBV (Zebra). The antibodies to these antigens were analyzed with fluorescent bead-based multiplex serology at the laboratory of German Cancer Research Center (DKFZ), Heidelberg (Germany). All EBV seroreactivities were expressed as medium fluorescence intensity values (MFI) for each of the four EBV antigens separately. The pre-defined MFI cut-offs of seropositivity for Zebra, EA-D, EBNA-1, and VCAp18 were 176, 367, 1500, and 1802, respectively^16^. The subject was classified EBV-seropositive when at least two of these four EBV antigens exceeded the cut-off value^16^. Altogether, 280 women had EBV IgG-serology data available at least from two follow-up visits. EBV serology outcomes were further grouped into (1) always seropositive, and (2) always seronegative during the 36-month follow-up. Seroconversion was defined as an event where an initially below the cut-off MFI value exceeded the cut-off point in the subsequent visit and showed more than a two-fold increase in the MFI values. For serological decay, the contrary was true, i.e., more than two-fold decrease in two subsequent MFI values, the latter also falling below the cut-off level.

Results of HPV serology in the FFHPV cohort have been published earlier^10^. In the present analyses, we used the data on serum antibodies to the major capsid protein (L1) of five HPV genotypes: 6, 11, 16, 18, and 45^10^. The cut-off for HPV seropositivity was the MFI value ≥ 200.

### Statistical analyses

Antibodies to each EBV antigen (Zebra, EA-D, EBNA-1, and VCAp18) were evaluated individually and with different combinations. Correlations in seroreactivity between the four antigens at each time point were assessed using Spearman’s rank correlation coefficient. Correlation strength was categorized as low (0.00-0.40), moderate (0.40–0.60), or high (0.60-1.00). Univariate unconditional logistic regression analysis was applied to evaluate associations between EBV-related variables, potential risk factors obtained from the study questionnaire, and HPV infection outcomes. In the first stage of analyses, elevated EBV antibody levels were compared across different covariates. For this purpose, MFI values of each EBV antigen were divided into tertiles where the highest tertile was compared to the combined low and middle tertiles among the covariates. The women with the lowest levels of EBV antibodies were used as the reference group. In the second stage of analysis, the possible associations between elevated EBV antibody levels and persistent oral and genital HPV infections were evaluated, with HPV-negative women used as the control group. All statistical analyses were performed using Stata 16.1 (STATA Corp., TX), and p-values were two-sided, and p-values < 0.05 were considered statistically significant.

## Results

Our results of EBV-serology were based on the antibodies detected to four synthetic peptides covering immunodominant epitopes of VCAp18, EBNA-1, EA-D, and Zebra. The majority, with 98.2% (*n* = 275) of the women, were seropositive with the definition that at least two EBV IgG antibodies were positive at the time points of sample collection (Table 1). Only 1.8% (*n* = 5) of the participants were EBV- seronegative throughout the follow-up period. Not a single case of EBV-seroconversion or EBV-antibody decay was observed. The highest mean level of EBV IgG antibodies was detected for VCAp18. Altogether, 97.9% (*n* = 274) of the participants were seropositive to this antigen, being the most common among the 4 tested antigens. When stratified by different EBV antigen combinations, 259 (92.5%) women were simultaneously seropositive to EBNA-1 and VCAp18, typically characteristic of past EBV infection, while 223 (79.4%) were seropositive to all four EBV antigens.

**Table 1:** Serological EBV- and antigen-specific outcomes of the women during the 36-month follow-up, with the Mean (SD) antibody levels (MFI) and their range.

EBV serology outcomes	*N* (%)	Mean	SD	Min	Max
Always EBV-positive*	275 (98.2)				
Zebra		4432.4	2586.1	10	15,345
EA-D		3925.2	2768.5	64	14,473
EBNA-1		6371.3	2595.3	95	14,689
VCAp18		8911.2	2722.4	557	17,609
Always EBV-negative	5 (1.8)				
Zebra		37.2	18.9	14	71
EA-D		125.7	96.7	1	324
EBNA-1		17.2	37.2	1	15
VCAp18		287.6	307.0	1	863
Antigen-specific outcomes**					
Always positive to antigen:					
Zebra	261 (93.2)	4653.2	2460.3	190	15,345
EA-D	245 (87.5)	4348.2	2631.5	378	14,473
EBNA-1	260 (92.9)	6676.7	2327.5	1609	14,689
VCAp18	274 (97.9)	8942.7	2681.5	1928	17,609
All 4 antigens are positive simultaneously	223 (79.4)				
Zebra		4817.0	2428.8	203	15,345
EA-D		4407.2	2670.2	378	14,473
EBNA-1		6735.0	2298.4	1609	14,689
VCAp18		8737.1	2621.7	1928	16,643
Zebra, EBNA-1, VCAp18 positive simultaneously	245 (87.5)				
Zebra		4667.4	2462.7	190	15,345
EBNA-1		6719.4	2307.9	1609	14,689
VCAp18		8995.3	2669.5	1928	17,609
EA-D, EBNA-1, VCAp18 positive simultaneously	230 (82.1)				
EA-D		4356.8	2659.8	378	14,473
EBNA-1		6722.2	2309.3	1609	14,689
VCAp18		8919.8	2672.7	1928	17,609
EBNA-1, VCAp18 positive simultaneously	259 (92.5)				
EBNA-1		6676.2	2329.8	1609	14,689
VCAp18		8964.1	2649.2	1928	17,609
Zebra, EA-D positive simultaneously	238 (85.0)				
Zebra		4802.7	2425.6	203	15,345
EA-D		4383.8	2643.8	378	14,473

*EBV seropositivity was defined by simultaneous seropositivity to two or more tested EBV proteins Zebra, EA-D, EBNA-1, and VCAp18. **Seropositivity to different combinations of EBV antigens.

**Fig. 1:**
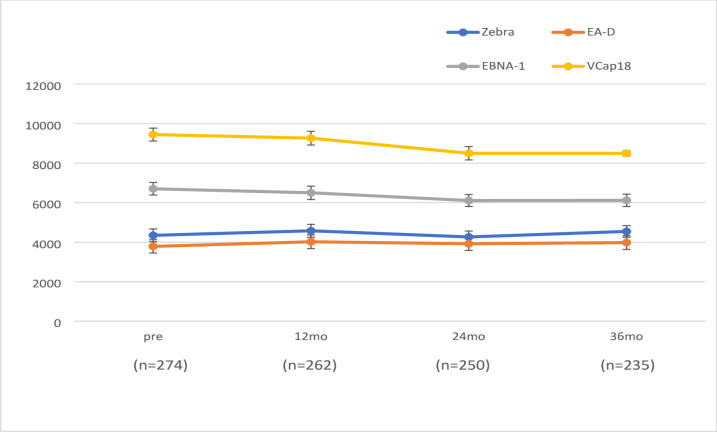
Mean levels of EBV IgG- antibodies to four different EBV antigens in women at different FU-visits. Error bars represent 95% confidence intervals (95%CI) of the mean antibody levels.

Figure 1 illustrates the fluctuation in mean MFI values of the four EBV antibodies among seropositive women during their 36-month follow-up. The levels of these antibodies remained consistently stable during the follow-up. This figure includes antibody levels from individuals classified as EBV-seropositive (by the defined criteria of 2/4 positive), and these mean MFI values also encompass samples categorized as EBV-seronegative. A comprehensive summary of all positive antigens, with their mean, median, and range of MFI values, across each time point, irrespective of the participant’s EBV serological status, can be seen in **Supplementary Table ****S1**. During the follow-up, at baseline, 12-month, 24-month, and 36-month visits, there were 20, 16, 16, and 12 EBNA-1 seronegative women, while the corresponding number of VCAp18 seronegative women was much less, 8, 5, 7, and 5, respectively. Among the tested antigens, EA-D-seronegativity was the most prevalent in women. At baseline, 39 women were seronegative to IgG EA-D, while at 12-, 24-, and 36-month visits, only 21, 22, and 13 women remained IgG-EA-D-seronegative. This indicates that a few women acquired EBV infection or the latent EBV infection reactivated. Interestingly, fewer women were ZEBRA-seronegative during the follow-up (19, 14, 16, and 10 women in the same order of the visits), even though seroresponse to ZEBRA is also a marker of EBV-reactivation.

Bivariate correlations of the antibody responses between the four EBV proteins are given in Table 2. There was a significant correlation between EBNA-1 and VCAp18 antibody levels for all follow-up visits (*p* < 0.001), indicating past EBV infection. EBNA-1 antibodies were also statistically significantly correlated with Zebra antibodies at all follow-up visits (*p* < 0.05), indicating EBV reactivation, as serum antibodies directed against Zebra are considered a good marker for viral reactivation. Similarly, EBNA-1 antibody levels showed a correlation with EA-D antibodies, but this correlation was statistically significant only at 12-month and 24-month visits (*p* < 0.05). EBV EA-D IgG antibodies are characteristic of recent EBV infection or reactivation. Accordingly, we found a significant correlation between Zebra and EA-D antibody levels throughout all follow-up visits (*p* < 0.001). EA-D-IgG antibodies showed a statistically significant correlation also with VCAp18, but only at 12 months and 24 months follow-up visits (*p* < 0.01), which might indicate a possible recent EBV infection. EBNA-1 IgG antibodies are produced later during EBV infection than IgG-antibodies to VCA.

**Table 2:** Bivariate correlations (Spearman’s rho) of the antibody levels between the four EBV antigens (Zebra, EA-D, EBNA, and VCAp18) at each follow-up visit.

	EBNA-1	Zebra	EA-D	VCAp18
Baseline				
EBNA-1	1.00			
Zebra	0.19*	1.00		
EA-D	0.10	0.44**	1.00	
VCAp18	0.36**	0.16*	0.09	1.00
12-months				
EBNA-1	1.00			
Zebra	0.20*	1.00		
EA-D	0.19*	0.53**	1.00	
VCAp18	0.53**	0.24**	0.23**	1.00
24-months				
EBNA-1	1.00			
Zebra	0.24**	1.00		
EA-D	0.18*	0.56**	1.00	
VCAp18	0.40**	0.23**	0.17*	1.00
36-months				
EBNA-1	1.00			
Zebra	0.18*	1.00		
EA-D	0.11	0.55**	1.00	
VCAp18	0.45**	0.15*	0.13	1.00

Statistically significant results: * *p* < 0.05; ** *p* < 0.001.

A variety of health and sexual behavior-related factors were tested as potential covariates of high EBV- antibody (highest tertile) levels as presented in Table 3. Reported atopy significantly increased the likelihood of high antibody levels to Zebra and VCAp18 levels, odd ratios (ORs) of 2.11 (95%CI 1.07–4.17) and 2.45 (95%CI 1.24–4.83), respectively. After adjusting for age and the number of sex partners, the results remained significant with the ORs of 2.06 (95% CI 1.01–4.18) and 2.49 (95% CI 1.22–5.08), respectively. Reported use of condoms sometimes or always seems to have a protective effect on high-level EBNA-1 antibodies. However, only the variable “using condoms sometimes” was statistically significantly protective, OR 0.16 (95% CI 0.03–0.84).

**Table 3:** Potential covariates associated with the highest tertile of the antibody levels to the four EBV antigens tested in univariate regression analysis.

Covariates	Zebra	EA-D	EBNA-1	VCAp18
	OR (95% CI)			
Number of sexual partners (*n* = 258):				
0–2	1.0	1.0	1.0	1.0
3–5	0.64 (0.32–1.29)	1.41 (0.72–2.78)	1.17 (0.59–2.33)	2.41 (1.17–4.99) *
6–10	1.35 (0.66–2.76)	1.00 (0.48–2.11)	1.16 (0.55–2.44)	1.71 (0.78–3.77)
≥ 10	0.56 (0.25–1.24)	0.57 (0.25–1.30)	0.68 (0.30–1.54)	1.57 (0.70–3.56)
Number of sexual partners before the age of 20 (*n* = 259):				
0–2	1.0	1.0	1.0	1.0
3–5	0.89 (0.49–1.62)	0.93 (0.52–1.66)	0.84 (0.47–1.51)	1.10 (0.61–1.96)
6–10	1.54 (0.74–3.21)	0.78 (0.36–1.68)	0.35 (0.14–0.85) *	0.61 (0.27–1.38)
≥ 10	0.25 (0.05–1.13)	0.34 (0.09–1.23)	0.84 (0.29–2.41)	0.73 (0.24–2.20)
First sexual intercourse above 16 (*n* = 259):	1.02 (0.60–1.72)	1.04 (0.62–1.75)	1.05 (0.62–1.77)	1.06 (0.63–1.79)
Practice of oral sex (*n* = 259):				
Never	1.0	1.0	1.0	1.0
Sometimes	1.92 (0.94–3.92)	1.86 (0.93–3.74)	1.32 (0.67–2.59)	1.18 (0.60–2.29)
Regularly	1.63 (0.60–4.38)	1.47 (0.55–3.90)	1.04 (0.39–2.76)	1.27 (0.50–3.26)
Reported history of oral contraceptive use (*n* = 259):	3.35 (0.96–11.66)	1.44 (0.54–3.81)	1.03 (0.40–2.63)	1.07 (0.42–2.73)
Reported use of condom (*n* = 154):				
Never	1.0	1.0	1.0	1.0
Sometimes	1.69 (0.33–8.71)	1.86 (0.64–5.42)	0.16 (0.03–0.84) *	1.46 (0.28–7.57)
Always	1.50 (0.24–9.44)	N/C	0.17 (0.03–1.05)	2.73 (0.44–16.75)
Anal sex (*n* = 259):				
Never	1.0	1.0	1.0	1.0
Sometimes	1.15 (0.60–2.23)	1.64 (0.86–3.10)	1.05 (0.54–2.05)	1.24 (0.64–2.37)
Regularly	1.05 (0.09–11.80)	1.05 (0.09–11.81)	1.05 (0.09–11.81)	N/C
Other reported sexually transmitted diseases (*n* = 275): **	0.70 (0.36–1.34)	**0.49 (0.24**–0.98) *	0.78 (0.41–1.48)	0.96 (0.51–1.80)
Reported allergies (*n* = 257):	0.62 (0.36–1.06)	1.04 (0.62–1.75)	0.95 (0.56–1.61)	0.89 (0.53–1.51)
Reported atopy (*n* = 251):	2.11 (1.07–4.17) *	1.60 (0.81–3.16)	1.43 (0.71–2.85)	2.45 (1.24–4.83) *
Miscarriages (*n* = 250):				
0	1.0	1.0	1.0	1.0
1	1.87 (0.84–4.15)	0.72 (0.30–1.70)	0.62 (0.25–1.53)	1.13 (0.49–2.57)
≥ 2	0.86 (0.16–4.57)	0.72 (0.14–3.78)	N/C	1.52 (0.33–6.98)
Parity (*n* = 259):				
0	1.0	1.0	1.0	1.0
1	0.47 (0.03–7.56)	0.54 (0.03–8.72)	0.41 (0.03–6.68)	0.44 (0.03–7.20)
2	0.64 (0.04–10.73)	0.51 (0.03–8.64)	0.59 (0.04–9.99)	0.59 (0.04–9.99)
≥ 3	0.13 (0.004–4.00)	0.29 (0.01–6.91)	2.00 (0.09–44.35)	1.25 0.06–26.87)

* *p* < 0.05 ** Other sexually transmitted diseases include chlamydia and genital herpes.

*-* MFI levels of antigen tertiles: Zebra (low = 176–4118, mid = 4119–6763, high = 6764–15345), EA-D (low = 367–3057, mid = 3058–6266, high = 6267–14473), EBNA-1 (low = 1500–6800, mid = 6801–9092, high = 9093–14689), VCAp18 (low = 1802–10143, mid = 10144–12570, high = 12571–17609).

The associations between EBV serology and persistent genital (*n* = 33) or oral HPV16 (*n* = 14) infections are summarized in Table 4. Persistent genital HPV16 infections were negatively associated with mid-elevated VCAp18 antibody levels, OR 0.18 (95% CI 0.05–0.71). This, however, did not remain significant with the highest VCAp18 antibody levels. No other associations between EBV antibody levels and persistent oral or genital HPV infection were found. A subgroup of 12 women who developed an incident cervical high-grade squamous intraepithelial lesion (HSIL) was also analyzed, but no significant association with EBV serology was found.

**Table 4:** The levels of antibodies (tertiles) to the four EBV antigens stratified by persistent oral and genital HPV16 infections and incident HSIL lesions.

EBV Antigen		Oral HPV16 persistence > 24mo (*n* = 14)	Genital HPV16 persistence > 24mo (*n* = 33)	Cervical HSIL progression (*n* = 12)
	MFI-tertiles	OR (95% CI)		
Zebra	low	1.0	1.0	1.0
	mid	1.54 (0.37–6.45)	1.75 (0.51–6.01)	0.44 (0.07–2.76)
	high	0.31 (0.05–1.94)	1.02 (0.28–3.68)	1.00 (0.21–4.81)
EA-D	low	1.0	1.0	1.0
	mid	1.17 (0.20–6.89)	0.50 (0.14–1.79)	1.20 (0.22–6.52)
	high	4.15 (0.86–19.92)	0.36 (0.10–1.39)	0.67 (0.13–3.44)
EBNA-1	low	1.0	1.0	1.0
	mid	0.57 (0.13–2.51)	0.43 (0.11–1.68)	0.42 (0.06–2.90)
	high	0.89 (0.19–4.11)	0.51 (0.13–1.97)	1.54 (0.30–7.87)
VCAp18	low	1.0	1.0	1.0
	mid	0.80 (0.17–3.67)	0.18 (0.05–0.71) *	0.57 (0.11–3.02)
	high	1.44 (0.32–6.49)	0.43 (0.12–1.53)	0.70 (0.13–3.72)
Antigen combinations				
Antigens positive simultaneously	≥ 2^a^	1.27 (0.11–15.23)	0.29 (0.05–1.55)	1.52 (0.14–16.33)
	all 4	1.45 (0.26–8.00)	1.25 (0.30–5.17)	1.63 (0.30–8.70)
	EBNA-1, VCAp18	0.55 (0.08–3.67)	N/C	N/C
	Zebra, EA-D	N/C	0.96 (0.22–4.25)	1.14 (0.21–6.26)
	Zebra, EBNA-1, VCAp18	0.75 (0.12–4.64)	2.07 (0.35–12.22)	1.66 (0.18–15.23)
	EBNA, VCAp18, EA-D	1.45 (0.26–8.00)	1.33 (0.27–6.51)	1.63 (0.30–8.70)

^a^ consists of subjects with 2 or more antigens in the highest antigen tertile at every timepoint throughout the 36-month FU.

Univariate regression analysis was applied to estimate ORs.

MFI levels of antigen tertiles: Zebra (low = 176–4118, mid = 4119–6763, high = 6764–15345), EA-D (low = 367–3057, mid = 3058–6266, high = 6267–14473), EBNA-1 (low = 1500–6800, mid = 6801–9092, high = 9093–14689), VCAp18 (low = 1802–10143, mid = 10144–12570, high = 12571–17609).

Zebra (transcriptional transactivator protein), EA-D (early antigen-diffuse), EBNA-1 (EBV nuclear antigen 1), VCAp18 (viral capsid antigen p18).

EBV antibody levels as related to HPV serology at baseline and HPV serological outcomes during the follow-up are depicted in Table 5. Elevated VCAp18 antibody levels were associated with low-risk HPV seropositivity, OR 1.92 (95% CI 1.06–3.45), but this significance disappeared when only the extremely high titers of VCAp18 were considered. No other associations between the HPV serology and EBV antibody levels were disclosed.

**Table 5:** The levels of antibodies (tertiles) to the four EBV antigens stratified by baseline HPV seropositivity and serological HPV outcomes.

		Baseline HPV seropositivity		HPV serology outcome during FU	
EBV antigen	MFI-tertiles	LR-HPV^b^	HR-HPV^c^	Always seronegative	Always high antibody levels
OR (95% CI)					
Zebra	low	1.0	1.0	1.0	1.0
	mid	0.64 (0.36–1.15)	0.77 (0.42–1.39)	0.83 (0.37–1.87)	0.72 (0.19–2.74)
	high	0.87 (0.49–1.56)	0.92 (0.51–1.66)	0.91 (0.41–2.01)	1.18 (0.32–4.42)
EA-D	low	1.0	1.0	1.0	1.0
	mid	1.14 (0.63–2.04)	0.92 (0.50–1.69)	0.99 (0.45–2.16)	0.93 (0.26–3.41)
	high	0.84 (0.47–1.50)	1.52 (0.84–2.74)	0.76 (0.33–1.73)	1.87 (0.44–7.85)
EBNA-1	low	1.0	1.0	1.0	1.0
	mid	0.60 (0.33–1.08)	0.71 (0.39–1.29)	0.75 (0.32–1.75)	1.56 (0.36–6.76)
	high	0.87 (0.48–1.57)	0.80 (0.44–1.44)	1.25 (0.57–2.70)	0.57 (0.14–2.32)
VCAp18	low	1.0	1.0	1.0	1.0
	mid	1.92 (1.06–3.45) *	0.94 (0.52–1.70)	0.33 (0.13–0.88)**	2.13 (0.48–9.50)
	high	1.04 (0.58–1.87)	0.91 (0.50–1.65)	1.30 (0.63–2.71)	0.42 (0.10−1.74)
At least 2 high EBV antigens^a^	yes	0.66 (0.31–1.43)	1.28 (0.59–2.79)	1.18 (0.42–3.28)	2.03 (0.47–8.68)

^a^ consists of subjects with 2 or more EBV antigens in the highest antigen tertile at every FU visit; ^b^ LR-HPV mothers (yes/no) n=182 (types 6 and 11); ^c^ HR-HPV mothers (yes/no) n=127 (types 16, 18 and 45); ^d^ HPV negative n=58; ^always^ high HPV levels n=36.

* p=0.032; **p=0.027.

Univariate regression analysis was applied to estimate ORs.

MFI levels of antigen tertials: Zebra (low = 176–4118, mid = 4119–6763, high = 6764–15345), EA-D (low = 367–3057, mid = 3058–6266, high = 6267–14473), EBNA-1 (low = 1500–6800, mid = 6801–9092, high = 9093–14689), VCAp18 (low = 1802–10143, mid = 10144–12570, high = 12571–17609).

## Discussion

To our knowledge, this is the first long-term study examining the relationships between EBV serology and HPV infection outcomes. Nearly all women were EBV-seropositive during the three-year follow-up, consistent with expected population prevalence. Self-reported history of atopy was associated with increased EBV antibody levels among these healthy young women. However, contrary to our hypothesis, no consistent associations were observed between elevated EBV antibody levels and longitudinal oral or genital HPV infection outcomes.

EBV is a widespread oncogenic virus, typically acquired during childhood or later in adolescence through transfer of saliva, resulting in mononucleosis or asymptomatic infection^1^. Seroprevalence increases with age, and most individuals seroconvert before the age of 30^1,2^. This pattern was also evident in our cohort, where 98% of participants were EBV-seropositive. Although sexual activity has been suggested as a risk factor for EBV acquisition in some studies, we found no associations between sexual behavior, e.g., the number of sexual partners, and EBV antibody levels.

Interestingly, we found that self-reported atopy was associated with higher EBV antibody levels. The association between atopy and allergic diseases and EBV seropositivity has been studied previously, with highly contradictory results. In some studies, EBV infection increased the risk of early atopy, while in some others, it was shown to be a protective factor against atopy and allergic diseases, and yet in others, no association between EBV and atopy was disclosed^17–20^. Our findings support the possibility of a link, although further studies are needed to clarify this relationship.

In this cohort, antibody levels against all four EBV antigens remained stable over the three-year follow-up period. Seropositivity to VCAp18 was the most prevalent. This observation may be explained by the fact that antibodies against VCAp18 typically emerge during the acute phase of infection and persist lifelong following primary infection^1^. Seropositivity to Zebra and EBNA-1 was observed at nearly comparable frequencies to VCAp18. The high prevalence of Zebra antibodies is particularly noteworthy, given its role in EBV reactivation and lytic infection, as well as its proposed involvement in EBV-driven oncogenesis and potential utility as a biomarker for nasopharyngeal carcinoma^13,21,22^. The IgG antibodies of the antigen EBNA-1 are usually not detectable during the early course of primary EBV infection and therefore indicate a past infection^23^. This could be the reason for the high seropositivity to the antigen observed in this cohort. Seropositivity was the least common to antigen EA-D, which could be explained by the fact that this is the prime antigen in the primary and acute EBV infections^24^. Some studies even state that antibody levels to EA-D would not be detectable after 3 to 4 months, but in some cases, EA-D antibodies can be detected years after the initial infection^24^. EA-D plays an important role in lytic replication^25^, and detection of EA-D antibodies can be used as a marker for the reactivation of the virus^23^.

The association between co-existent mucosal EBV and HPV prevalence has been reported in some previous studies to increase concurrently with cervical lesion severity from normal to HSIL and further in cervical cancer^12,26^. Additionally, the overall simultaneous presence of EBV and HPV DNA in oral (OSCC) and oropharyngeal squamous cell carcinomas (OPSCC) has been estimated to be 11.9%, with the highest DNA copresence being 34.7% for OSCC and 23.4% for OPSCC^27^. Thus, the co-existence of EBV and HPV DNA in carcinomas has been documented, but the true impact of these viruses on each other’s activity remains to be better understood.

The modes of cooperation between EBV and HPV in cervical cancer development could be by two different mechanisms: (1) infecting epithelial cells, possibly synergizing with HR-HPV (a direct mechanism), and (2) infecting tissue-infiltrating lymphocytes and generating local immunosuppression (an indirect mechanism).

Most previous studies have focused only on the simultaneous detection of EBV and HPV DNA while largely overlooking the humoral immune response to these viruses. Some investigations have assessed serum IgG antibodies against EBV antigens, including VCA, EBNA-1, and EA-D, in relation to the progression of cervical intraepithelial neoplasia (CIN). However, no significant differences in EBV serological profiles have been observed between cases with progressive and non-progressive CIN^26^. The same research group also evaluated the role of these EBV-specific antibodies in patients with OPSCC, again reporting no significant associations^28^. To the best of our knowledge, there are currently no other long-term longitudinal studies examining EBV serology in relation to HPV infection outcomes. The present study is therefore the first to assess antibodies against four distinct EBV antigens in relation to both oral and genital HPV infection outcomes in a prospective cohort of women, thereby providing more insight into the potential interplay between these two oncogenic viruses. Unfortunately, the limited number of seronegative participants precluded meaningful comparisons between EBV-seropositive and -seronegative individuals, and analyses were instead focused on variation in antibody levels among seropositive participants.

It has been suggested that consistent, and especially high titers of EBV antibodies could impact EBV-related malignancies^1,29^, which prompted us to hypothesize that elevated EBV antibodies could affect the natural history of HPV infections. However, when comparing EBV serology with HPV infection outcomes, only VCAp18 antibody levels showed a negative association with persistent genital HR-HPV infections (*n* = 33). No correlations were found between EBV serology and oral HPV infections, which was unexpected given EBV’s lifelong presence in the oral cavity and its shedding pattern, distinct from HPV. Ultimately, we could not find any firm evidence to support our hypothesis, as no other associations were observed between elevated EBV antibody levels and the clinical outcomes of either oral or genital HR-HPV infections.

The strengths of our study include a three-year follow-up period, which, to our knowledge, is the first to examine EBV serology in relation to HPV serology and HPV DNA in genital and oral sites. In addition, we had a relatively large sample size compared to previous studies. Unlike earlier research, which focused on EBV and HPV DNA in malignant lesions, our study explored EBV serology’s effects on the natural history of HPV infections, before oncogenesis.

Several limitations should also be considered. First, data on local EBV DNA and the co-localization of EBV and HPV within the same cells. Reliance on EBV IgG serology alone may be suboptimal, particularly given the high seroprevalence observed in the study population. Furthermore, IgM antibodies were not analyzed, precluding the identification and timing of primary EBV infection. In addition, mucosal IgA antibodies were not measured, limiting assessment of local immune responses. Participant loss during follow-up also led to missing blood samples, potentially overlooking fluctuations in EBV antibodies. Nevertheless, the majority of participants (78.2%) had complete data from all four time points, while only 8.6% had data from two time points. Finally, the study’s focus on healthy young women limits our ability to generalize EBV’s impact on the natural history of HPV over a short period (3 years).

## Conclusions

Nearly all women were EBV-seropositive, as expected. Self-reported history of atopy was positively associated with increased EBV antibody levels among these healthy young women. Contrary to our hypothesis, no clear associations between elevated EBV antibody levels and longitudinal oral and genital HPV outcomes could be found. Still, earlier studies have shown some connection between the viruses, and further studies are needed to explore the association between HPV and EBV more thoroughly, particularly in relation to persistent HPV infections and the potential impact on HPV-associated malignancies.

## Supplementary Information

Below is the link to the electronic supplementary material.

Supplementary Material 1

## Data Availability

The data generated in this study are available upon request from the senior author [KL].
