# Supplementary material for: Epstein-Barr virus serology in relation to the outcomes of oral and genital human papillomavirus infections in women during 3-year follow-up
Source: Sci Rep. 2026 May 29;16:24626. doi: 10.1038/s41598-026-54544-7 (PMC13454423; doi:10.1038/s41598-026-54544-7)
Supplement: Supplementary file 1 [file 41598_2026_54544_MOESM1_ESM.docx]

**Supplementary material**

| **SUPPLEMENTARY TABLE 1**  The Mean (SD) levels and range of all positive EBV IgG antigens throughout the follow-up period | | | | | | | | |
| --- | --- | --- | --- | --- | --- | --- | --- | --- |
|  | **MFI** | | | | | | | |
| **EBV antigen** | | **FU-visit** | **N of EBV- seropositive** | **Mean** | **SD** | **Median** | **Min** | **Max** |
|  | |  |  |  |  |  |  |  |
| **Zebra**  (cut-off MFI 176) | | |  |  |  |  |  |  |
|  | | baseline | 299 | 4417.5 | 2659.8 | 4657 | 201 | 11710 |
|  | | 12mo | 253 | 4782.8 | 2609.4 | 4676 | 182 | 15345 |
|  | | 24mo | 241 | 4421.9 | 2322.9 | 4376 | 178 | 11812 |
|  | | 36mo | 231 | 4662.1 | 2407.1 | 4488 | 197 | 12112 |
| **EA-D**  (cut-off MFI 367) | | |  |  |  |  |  |  |
|  | | baseline | 279 | 4081.9 | 2727.3 | 3658 | 383 | 13860 |
|  | | 12mo | 246 | 4284.4 | 2742.3 | 3670.5 | 384 | 12526 |
|  | | 24mo | 235 | 4157.6 | 2627.0 | 3563 | 378 | 11968 |
|  | | 36mo | 228 | 4121.9 | 2625.4 | 3692 | 378 | 14473 |
| **EBNA-1**  (cut-off MFI 1500) | | |  |  |  |  |  |  |
|  | | baseline | 298 | 6983.3 | 2450.0 | 6892 | 1600 | 14689 |
|  | | 12mo | 251 | 6810.7 | 2571.0 | 6666 | 1609 | 15103 |
|  | | 24mo | 241 | 6313.0 | 2211.5 | 6186 | 1643 | 13345 |
|  | | 36mo | 229 | 6316.2 | 2242.1 | 5935 | 1503 | 13728 |
| **VCAp18**  (cut-off MFI 1802) | |  |  |  |  |  |  |  |
|  | | baseline | 310 | 9603.4 | 2727.8 | 9446.5 | 1928 | 17411 |
|  | | 12mo | 262 | 9323.8 | 2836.9 | 8992.5 | 2686 | 17609 |
|  | | 24mo | 250 | 8530.1 | 2656.3 | 8167.5 | 1989 | 17115 |
|  | | 36mo | 236 | 8361.6 | 2343.4 | 8135 | 2297 | 15182 |

Number of tested women by visit: baseline (n=318), 12mo (n=267), 24mo(n=257), 36mo(n=241)
